# Efficacy of Plasma-Treated Water against Salmonella Typhimurium: Antibacterial Activity, Inhibition of Invasion, and Biofilm Disruption

**DOI:** 10.3390/antibiotics12091371

**Published:** 2023-08-27

**Authors:** Adrian Abdo, Andrea McWhorter, Daniel Hasse, Thomas Schmitt-John, Katharina Richter

**Affiliations:** 1 Richter Lab, Department of Surgery, Basil Hetzel Institute for Translational Health Research, The Queen Elizabeth Hospital, University of Adelaide, Woodville, SA 5011, Australia; adrian.abdo@adelaide.edu.au; 2 School of Animal and Veterinary Sciences, University of Adelaide, Roseworthy, SA 5371, Australia; andrea.mcwhorter@adelaide.edu.au; 3 Plasmatreat GmbH, 33803 Steinhagen, Germany; 4 Institute for Photonics and Advanced Sensing, University of Adelaide, Adelaide, SA 5005, Australia

**Keywords:** *Salmonella*, plasma-treated water, cold plasma technology, non-thermal plasma, biofilm, bacterial invasion, sanitizer, decontamination, food safety

## Abstract

Plasma-treated water (PTW) has emerged as a potential sanitizing agent. This study evaluated antibacterial activity, inhibition of invasion, and biofilm disruption effects of PTW against *Salmonella* Typhimurium. Minimum inhibitory concentrations (MICs) and minimum bactericidal concentrations (MBCs) were determined for different PTW types. Time-kill assays were conducted to assess bactericidal effects, while polarized Caco-2 cells were used to evaluate invasion inhibition. Biofilm formation and cell viability were examined following PTW treatment using *Salmonella* Typhimurium isolates, while biofilm disruption and regrowth prevention were investigated using the Bioflux system. PTW exhibited antibacterial activity against all Salmonella Typhimurium isolates, with MICs of 25% for PTW1 and PTW2, and 50% for PTW3, PTW4, and PTW5. MBCs of 50% in media were observed for all PTW types. Undiluted PTW1 and PTW2 showed the highest bactericidal capacity, significantly reduced *Salmonella* viability, and completely inhibited bacterial invasion, while PTW3 and PTW5 also showed significant invasion reduction. Bioflux experiments confirmed the eradication of biofilms by PTW1 and PTW2, with no regrowth observed 72 h after PTW was removed. PTW demonstrated significant antibacterial activity, inhibition of invasion, biofilm disruption, and reduction of bacterial viability against *Salmonella* Typhimurium. This highlights PTW’s potential as an effective sanitizer for reducing *Salmonella* contaminations.

## 1. Introduction

In 2010, the World Health Organization’s Foodborne Disease Burden Epidemiology Reference Group estimated that 600 million incidents of disease and 420,000 deaths world-wide could be linked with foodborne disease [1]. Of those, over 78 million incidents of disease and more than 59,000 mortalities were caused by infection with non-typhoidal *Salmonella enterica* serotypes [1]. The genus *Salmonella* comprises two species, *Salmonella enterica* and *Salmonella bongori* [2,3]. The species, *Salmonella enterica*, comprises six subspecies, but members of *Salmonella enterica* subspecies I (*Salmonella enterica* subspecies *enterica*) are most commonly linked with human disease [1]. Symptoms of *Salmonella* infection generally include self-limiting diarrhea, cramping, and fever.

*Salmonella* is an enteric bacterial pathogen that routinely establishes persistent infection in the gastrointestinal system of food animals. Thus, contamination of the food supply chain represents a significant public health risk. A wide range of food items have been identified as potential sources of *Salmonella*, including milk, red meat, pork, seafood, poultry meat, eggs, fruits, vegetables, grains, and low moisture foods such as chocolate and peanut butter [4]. Therefore, methods to mitigate bacterial loads on or in food are of vital importance.

Many countries have established standardized protocols to minimize bacteria in the food supply chain [5,6,7], using food grade sanitizers, such as chlorine or quaternary ammonium compounds. However, tolerance to sublethal concentrations of sanitizers is emerging [8]. *Salmonella* has been shown to become tolerant to many bacterial control measures, including sanitizers and heat treatments used in different food industries. In addition, many countries do not permit the use of particular chemicals during food processing [9]. The emergence of multi-drug resistant *Salmonella* is also a significant threat to the safety of food and is an important public health risk [10]. Many *Salmonella* serotypes also possess the capacity to form biofilms, enhancing their ability to persist in the food supply chain [11]. Thus, there is a clear need for novel measures to control foodborne pathogens in the food supply chain.

Non-thermal or cold plasma technologies are emerging as industrial chemical-free disinfection methods to control pathogens in water [12] and food [13]. Plasma treatment of water produces a range of reactive oxygen and nitrogen species (RONS), including atomic oxygen (O), hydroxyl radicals (^•^OH), ozone (O_3_), hydrogen peroxide (H_2_O_2_), nitric oxide (NO^•^), nitrites (NO_2_^−^), nitrates (NO_3_^−^), and peroxynitrite (ONOO^−^). The concentration of ions and RONS in water reduces the pH and increases the electrical conductivity (S/m) and oxidoreductive potential (ORP) [14].

The antimicrobial properties of RONS have been well described and cold plasma-treated water (PTW) has been shown to be effective at killing a wide range of bacterial species [15,16]. The physiochemical properties of PTW and the concentration of each RONS, however, depends on the type of plasma device used, the carrier gas (e.g., air, helium, argon, or helium with oxygen), power, electromagnetic frequency, cold plasma exposure time, as well as the environmental conditions under which PTW is generated [17].

World-wide, the *Salmonella enterica* subspecies *enterica* (*Salmonella*) Typhimurium serotype is one of the predominant causes of foodborne bacterial gastrointestinal disease [18]. This study investigated the bactericidal capacity of 5 PTW types with different physiochemical properties on *Salmonella* Typhimurium. The aim of the present study is to validate the use of PTW as a food sanitizer with potential applications in a wide range of situations in the food supply chain to control foodborne pathogens.

## 2. Results

### 2.1. Minimum Inhibitory and Minimum Bactericidal Concentrations of Plasma-Treated Water

All PTW types exhibited antibacterial activity against the four *Salmonella* Typhimurium isolates included in this study. A minimum inhibitory concentration (MIC) of 25% was observed for types PTW1 and PTW2, while PTW3, PTW4, and PTW5 exhibited an MIC of 50%. The minimum bactericidal concentration (MBC) for all PTW types was 50% diluted in media.

### 2.2. Time-Dependent Killing of Salmonella Typhimurium by Plasma-Treated Water

**Figure 1:**
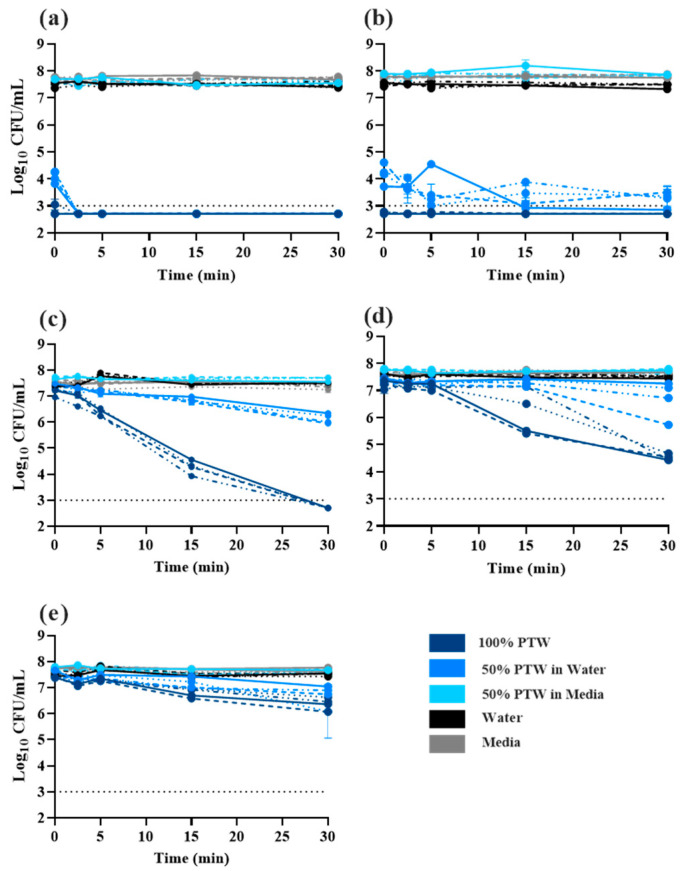
Time-dependent killing of *Salmonella* Typhimurium expressed as Log_10_ colony forming unit (CFU)/mL. Salmonella Typhimurium isolates R2 (dashed line), R5 (dotted line), R7 (dash and dotted line), and ATCC14028 (solid line) were suspended in either (**a**) PTW1, (**b**) PTW2, (**c**) PTW3, (**d**) PTW4, or (**e**) PTW5. Bacterial culturability was evaluated over a 30 min period. The black dotted line at 3 Log_10_ CFU/mL indicates the limit of detection.

Time-kill curves were performed to assess the bactericidal effects of different preparations of PTW. PTW types were diluted in media or water to observe the buffering effect of organic material on bactericidal properties. PTW1 and PTW2 exhibited the most effective bactericidal capacity. For 100% PTW1 and PTW2, all four *Salmonella* Typhimurium isolates were not culturable immediately following exposure (Figure 1a,b). For PTW1 and PTW2, dilution to 50% in water did not significantly affect the bactericidal capacity. Dilution of all PTW to 50% in media, however, substantially affected the bactericidal effect. Bacterial loads observed in diluted PTW treatments did not differ significantly from water or media only controls (Figure 1a–e). Undiluted PTW3 (Figure 1c) exhibited bacterial killing, but the kinetics were slow compared with PTW1 and PTW2. While there was greater than 3.0 log reduction of *Salmonella*, culturable bacteria were observed over the entire time-course. A similar response was observed for undiluted PTW4 (Figure 1d). Dilution of PTW3 and PTW4 in either media or water greatly impacted *Salmonella* killing over time. PTW5 did not exhibit significant bacterial killing over the 30 min period of testing (Figure 1e).

### 2.3. Effects of Plasma-Treated Water on Bacterial Invasion into Intestinal Epithelial Cells

**Figure 2:**
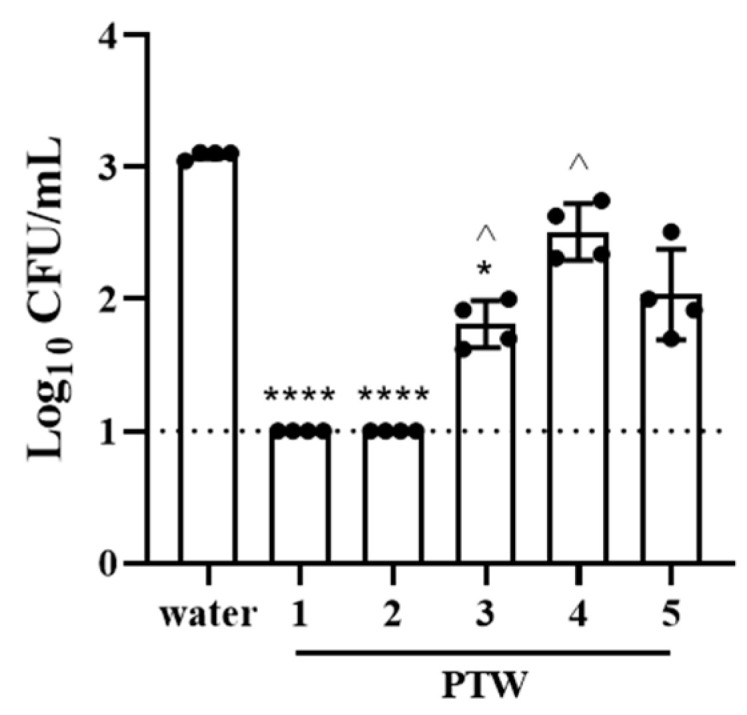
Treatment with PTW significantly reduced the invasive capacity of *Salmonella* Typhimurium. Data from 4 replicates, +/− standard deviation. Dotted line designates the limit of detection of the assay. * and **** represent *p* < 0.05 and <0.0001 compared to water group, respectively. ^ represents *p* < 0.05 compared to PTW1 and PTW2.

The capacity to invade eukaryotic cells is an important aspect of *Salmonella* virulence. The effect PTW had on *Salmonella* invasive capacity was investigated using polarized Caco-2 cells. Data were log_10_ transformed and are presented as mean Log_10_ CFU/mL ± standard deviation. No significant difference in invasive capacity was observed between strains 14028 and R2. Thus, to focus on the effect PTW treatment, data were combined. Compared with water, PTW1 and PTW2 significantly reduced bacterial invasion to below detectable levels, where no culturable bacteria were observed for any dilution of cell homogenate, eliminating the invasive capacity of *Salmonella* Typhimurium. Additionally, a significant 1.2 log reduction was also seen with PTW3 (Figure 2). Inhibition of bacterial invasion by PTW1 and PTW2 was also significantly greater than PTW3 and PTW4.

### 2.4. Biofilm Cell Viability

**Figure 3:**
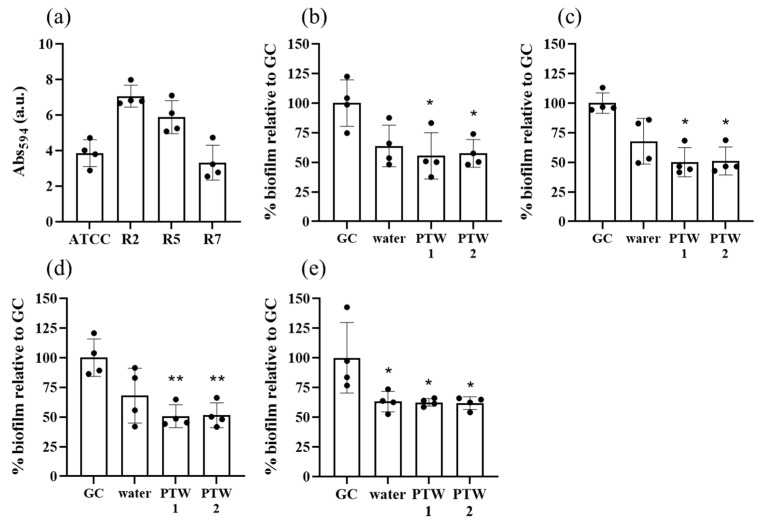
Effect of PTW1 and PTW2 on *Salmonella* biofilms. The biofilm-forming capacity of the four Salmonella Typhimurium isolates (R2, R5, R7, and ATCC14028) was compared (**a**). Biofilm formed by (**b**) ATCC 14028, (**c**) R2, (**d**) R5, and (**e**) R7 were all significantly reduced following exposure to PTW1 or PTW2 compared to the growth control (GC). * and ** represent *p* < 0.05 and *p* < 0.01. Data from 4 replicates, +/− standard deviation.

Based on results obtained from the time-kill and cell invasion experiment, the efficacy of PTW1 and PTW2 at controlling *Salmonella* biofilms was explored. The biofilm-forming capacity of the four different *Salmonella* Typhimurium isolates varied considerably between strains, with ATCC 14028 and R7 producing between 40–50% less biofilm than the R2 and R5 isolates (Figure 3a). The biofilm mass was significantly reduced in all four *Salmonella* Typhimurium isolates by treatment with PTW1 or PTW2 compared to the growth control (Figure 3b–e). No significant difference was observed between the water control and PTW treatments.

**Figure 4:**
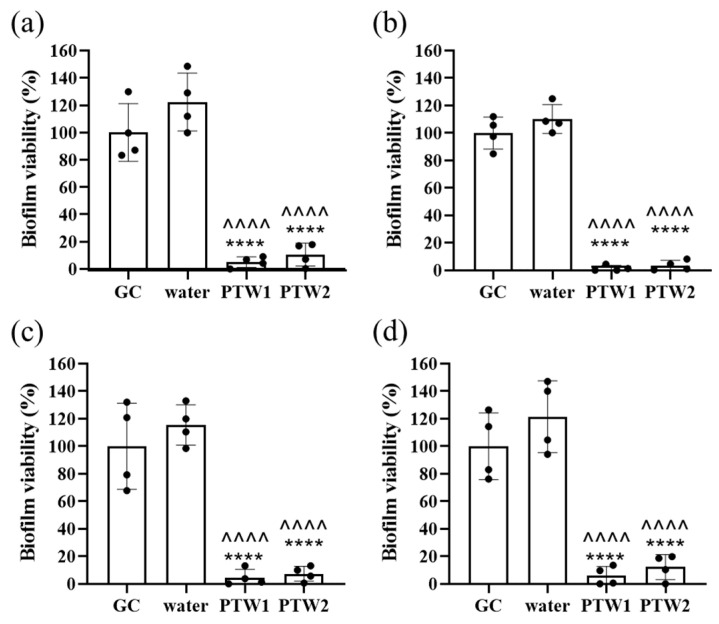
Cell viability of *Salmonella* Typhimurium in biofilms following PTW treatment. Following treatment with PTW1 and PTW2, the viability of ATCC 14028 (**a**), R2 (**b**), R5 (**c**), and R7 (**d**) were significantly reduced compared with water and growth controls (GC). **** represents *p* < 0.0001 compared to growth control and ^^^^ represents *p* < 0.0001 compared to water group. Data from 4 replicates, +/− standard deviation.

The antibiofilm activity of PTW types, PTW1 and PTW2, was also investigated by determining the viability of *Salmonella* Typhimurium in biofilms following treatment. The viability of *Salmonella* Typhimurium 14028, R2, R5, and R7 were all significantly reduced after a 5-min treatment with either PTW1 or PTW2. Cell viability was significantly reduced in PTW biofilms compared with the growth and water controls (Figure 4a–d). No significant difference was detected between the growth or water controls and no differences were observed between PTW1 and PTW2.

### 2.5. Bioflux

**Figure 5:**
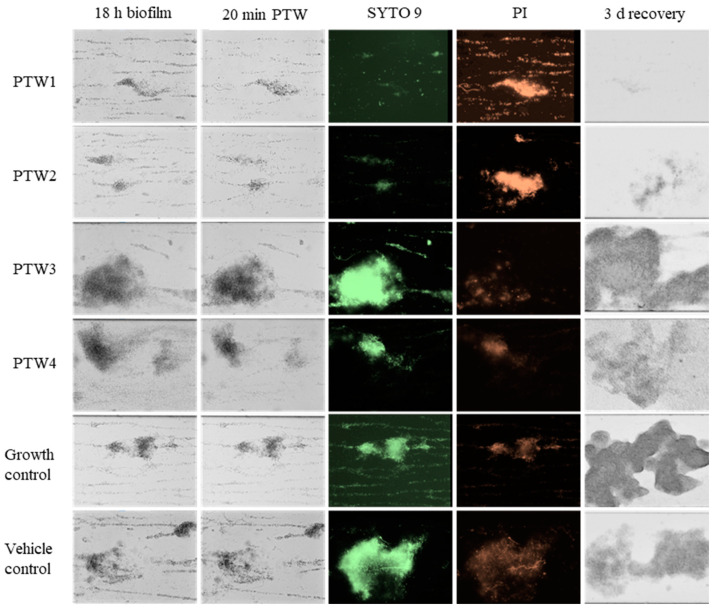
Visualization of *Salmonella* Typhimurium ATCC 14028 biofilm formation over 18 h, effects of treatment with PTW types, media (growth control) or RO water (vehicle control), and bacterial recovery 3 days post-treatment using the Bioflux system. Images were taken at 20× magnification. Green staining (SYTO 9) represents viable bacteria, red staining (propidium iodide; PI) represents dead bacteria.

*Salmonella* Typhimurium ATCC 14028 formed biofilms when grown in half-strength Luria Bertani broth without NaCl under steady nutrient flow at 25 °C in the Bioflux system (Figure 5, first column). Treatment with PTW1 or PTW2 resulted in shrinking of biofilm clusters and partial detachment of biofilms within 5 min of treatment (Figure 5, second column; Supplementary Materials Video S1). When exposed to Live/Dead staining, microscopy confirmed that the majority of biofilms were dead (red dye) with few viable bacteria left (green dye) (Figure 5, third and fourth column). In contrast, the majority of biofilms that were only exposed to media (growth control), vehicle control (water), PTW3 or PTW4 were viable as indicated by green staining and the absence of red staining. After another 3 days of incubation at 25 °C without flow, no regrowth of biofilms was observed in the PTW1 or PTW2 treatment group compared to bacterial growth observed in all other groups (Figure 5, fifth column).

## 3. Discussion

Salmonellosis in humans can occur following ingestion of >10^4^ *Salmonella* cells from contaminated food or water [19], but this may be impacted by host factors, including age, immunocompetency, and the type of food ingested [20]. Consequently, the minimum allowable number of *Salmonella* detected in food items is low. The International Microbiological Criteria for safe food defines food as ‘satisfactory’ if *Salmonella* is “not detected in 25 g” [21]. Current food decontamination methods frequently include the use of chlorine, quaternary ammonium compounds, or other food grade sanitizers. Recent work, however, has demonstrated that *Salmonella* isolates can be highly variable in their response to chlorine and that under certain conditions, bacteria recover following exposure [22]. Thus, there is a clear need for developing novel, more efficacious food sanitizers.

PTW has been previously shown to have bactericidal properties for Gram-positive [23] and Gram-negative [24] bacterial species. The production of PTW results in the generation of RONS, which is linked with a reduction of pH and known to have antimicrobial properties. Most studies, however, have only investigated a single plasma-treated water preparation. For *Salmonella*, a pH < 3.5 is considered lethal [25,26]. Thus, the present study evaluated different plasma-treated water preparations that resulted in different concentrations of RONS and pH.

MIC experiments conducted in the present study demonstrated that PTW1 and PTW2 had an MIC of 25%; all other PTW were 50%. MBC for all isolates was 50%. These results suggested that the presence of organic material in the media could potentially affect the efficacy of PTW. RONS species are not stable and react quickly upon exposure to organic material, including membrane lipids, proteins, nucleotides, and antioxidant molecules, as well as being prone to metabolism by antioxidant enzymes [27].

Time-kill curves were conducted to evaluate the efficacy of PTW killing of *Salmonella*. Undiluted PTW1 and PTW2 were highly effective at inactivating planktonic *Salmonella*; no culturable bacteria were observed after 2.5 min of exposure. Bacteria treated with PTW types 3 and 4 also exhibited killing over time. PTW3 had a 5-log reduction over all and by the end of the experiment, no culturable bacteria were observed. Significant variability of the response of *Salmonella* isolates to undiluted PTW4. Undiluted PTW5 exhibited the worst bactericidal effect. Only a 1 log reduction was observed. Dilution to 50% in water had no effect on PTW1 or PTW2, but substantially impacted the other PTW types, suggesting that there might be a scavenging effect of the RONS. Dilution to 50% in media had significant effects for all PTW types. No effects on bacteria were observed and the bacterial loads recovered did not differ for water or media controls. These results highlight the importance of both pH and the concentration of RONS for antibacterial activity. The proposed usage of PTW would be in the form of a wash, dip, or spray which would maximize the delivery of undiluted PTW.

Invasion is an important aspect of *Salmonella* pathogenicity and is linked with its capacity to cause disease [28]. The invasive capacity of *Salmonella* Typhimurium into cultured human intestinal epithelial cells (Caco-2) post-exposure to different PTW types was investigated. All PTW types significantly reduced bacterial invasion into Caco-2 cells compared with the control treatment. Treatment with PTW1 and PTW2, however, completely inhibited *Salmonella* invasion; no bacteria were recovered from the Caco-2 lysate. Cell viability of planktonic bacteria treated with PTW was not evaluated in this study. *Salmonella* cells in biofilm treated with PTW1 or PTW2 exhibited very low viability. Thus, it is not surprising that no invasiveness was observed for *Salmonella* Typhimurium following exposure to either PTW1 or PTW2.

The efficacy of PTW1 and PTW2 in time-kill and invasion experiments lead to the focus on their capacity to eradicate biofilms. Biofilms are ubiquitous in the food industry. *Salmonella* is able to form biofilms on eggs [29], as well as on food service and processing equipment. PTW was evaluated as a potential method to mitigate biofilms as a potential surface disinfectant. PTW1 and PTW2 exhibited a significant reduction in *Salmonella* biofilm compared with water and growth controls. The viability of remaining bacteria in the biofilm was extremely low. These results demonstrate the surface decontamination capacity of these two PTW preparations.

PTW1 and PTW2 effectively disrupted Salmonella biofilms within a short exposure time of 5 min, while Live/Dead staining further confirmed the efficacy of PTW1 and PTW2 in inducing cell death inside the biofilm, which is in line with current literature [30]. Treatment with PTW1 or PTW2 also resulted in the shrinking and partial detachment of biofilm clusters. After three days of incubation, no regrowth of biofilms was observed in the PTW1 or PTW2 treatment group. These findings highlight the potent biofilm-disrupting activity of PTW1 and PTW2 against Salmonella Typhimurium. The rapid and effective disruption of biofilms and prevention of regrowth are crucial in combating *Salmonella* contamination in food and reducing the risk of food poisoning for public health. The ability of PTW1 and PTW2 to rapidly disrupt biofilms and prevent regrowth is particularly noteworthy, as biofilm eradication is challenging due to increased resistance mechanisms and altered phenotypes of biofilm-associated bacteria.

Further research is necessary to explore the specific mechanisms of action underlying PTW1 and PTW2’s biofilm-disrupting effects [16], as well as to evaluate long-term efficacy, safety, and feasibility in real-world applications. Although the antibacterial and antibiofilm mechanisms of PTW on *Salmonella* are scarce, useful insight can be obtained by the effects on other biofilm species. *E. faecalis* biofilms exposed to PTW experienced prolonged downregulation of quorum sensing-related genes that impaired further biofilm formation [31]. Similarly, in addition to PTW achieving a >4 Log_10_CFU reduction of *Staphylococcus aureus* biofilm, surviving *S. aureus* had a 30% reduction in biofilm regrowth after a single exposure [32]. It is still contentious with regard to what components of PTW confer antimicrobial activity. The selective scavenging of major RONS H_2_O_2_, ^•^OH, O_3_, or NO from PTW did not significantly diminish the antimicrobial efficacy of PTW against *E. coli* or *Listeria innocua* [33], supporting the notion that antimicrobial effects of PTW are attributed to its complex chemistry. This complex chemistry also contributes to reducing its pH. Studies that experimentally simulated the pH effects of PTW using HCl or HNO_3_ had similar antimicrobial effects to PTW [34], underpinning pH and ORP as indicative properties of the antimicrobial effect of PTW [35,36]. Similar studies warrant being replicated in different serotypes of Salmonella biofilms over multiple exposures to PTW. Additionally, comparative studies with other antimicrobial agents and assessment of PTW’s effects on different Salmonella strains and biofilm types would contribute to a more comprehensive understanding of its potential in biofilm control strategies.

## 4. Materials and Methods

### 4.1. Chemicals and Reagents

All consumables and reagents were purchased from Thermo Fisher Scientific (Adelaide, Australia), unless otherwise stated. All aqueous reagents (except PTW) were prepared with ultrapure water (18.2 MΩ-cm) filtered through an Arium Pro water system (Sartorius, Göttingen, Germany).

### 4.2. Plasma-Treated Water Types

Five different preparations of PTW (Plasmatreat, Steinhagen, Germany) were tested for antimicrobial activity against *Salmonella*. PTWs were either prepared using Arc discharge plasma or dielectric barrier discharge plasma. The five types and their physiochemical properties are listed in Table 1.

### 4.3. Bacterial Strains

Three field *Salmonella* Typhimurium isolates (designated R2, R5, and R7) were selected for use in these experiments. These isolates were cultured from whole chicken carcasses obtained from commercial meat processors prior to any sanitizer treatment. Isolates were characterized at the *Salmonella* Reference Laboratory (Adelaide, Australia). The American Type Culture Collection (ATCC) *Salmonella* Typhimurium strain, 14028, was included as a serotype control. All cultures were maintained in a 1:1 mixture of Brain Heart Infusion (BHI) broth and glycerol and stored at −80 °C. Prior to experiments, bacteria were resuscitated on nutrient agar.

### 4.4. Minimum Inhibitory and Minimum Bactericidal Concentrations of PTW Types

All *Salmonella* Typhimurium isolates were streaked out onto nutrient agar and incubated at 37 °C for 18 h prior to the MIC experiment. The MIC of the PTW preparations was determined using the broth dilution method in Mueller Hinton broth (MHB) following the Clinical and Laboratory Standard Institute guidelines with a few modifications [37]. To prepare the challenge plate for each PTW type, 90 µL of cation-adjusted MHB was added to each well of a round bottom microtiter plate. Ninety µL of PTW was added to column 1 and serial two-fold dilutions were prepared for columns 1–10, generating a dilution series from 50% to 0.095%. Colonies were added to sterile 0.9% saline to generate a 0.5 McFarland Standard Unit (MFU) bacterial suspension. This suspension was then diluted 1:20 in sterile 0.9% saline to generate a bacterial inoculum concentration of 10^7^ colony forming units (CFU)/mL. Ten µL of the *Salmonella* suspensions was added to columns 1–11 (final concentration 10^6^ CFU/mL). Growth and sterility/negative controls were included. MIC plates were incubated for 18 h at 37 °C. Plates were read against a dark non-reflective surface. The MIC was the lowest concentration of PTW that inhibited bacterial growth in microtiter plates. To determine the minimum bactericidal (MBC), 10 µL from each well was plated onto nutrient agar. Plates were incubated for 18 h at 37 °C. The MBC was the lowest concentration of PTW that prevented bacterial regrowth on agar plates. Both MIC and MBC experiments were conducted in duplicate over three independent experiments.

### 4.5. Time-Dependent Killing Assay

Time-kill experiments were performed to characterize the susceptibility of *Salmonella* Typhimurium to the five types of PTW. Based on results obtained from MIC and MBC experiments, *Salmonella* Typhimurium isolates R2, R5, R7, and ATCC 14028 were treated with either 100% PTW or 50% PTW diluted in either sterile RO water or nutrient broth. *Salmonella* Typhimurium colonies were added to sterile 0.9% saline to generate an 0.5 MFU bacterial suspension. Ten μL of this suspension was added into 990 μL of PTW treatment to obtain 10^6^ CFU/mL.

Experiments were conducted at 25 °C. Bacterial counts were determined after 0, 2.5, 5, 15, and 30 min of exposure. Serial tenfold dilutions in 0.9% saline were prepared and 10 μL of each dilution was drop plated onto nutrient agar and incubated aerobically at 37 °C for 18 h. Colonies were enumerated and reported as CFU/mL. Each treatment group contained three biological replicates over three independent experiments.

### 4.6. Salmonella Invasion into Culture Human Intestinal Epithelial Cells (Caco-2)

The immortalized human colorectal adenocarcinoma cell line, Caco-2 (ATCC HTB-37), was selected as the relevant cell type to model an intestinal epithelial barrier to evaluate the invasive capacity of *Salmonella* post exposure to PTW. Prior to the experiments, cells were cultured in Dulbecco’s Modified Eagle media (DMEM) containing 4 mM glutamine, glucose, 10% (*v*/*v*) fetal bovine serum (FBS), 100 U/mL penicillin, and 100 µg/mL streptomycin at 37 °C with 5% CO_2_. Caco-2 cells were expanded in growth media and then sub-cultured into wells of a 48-well tissue culture tray at a concentration of 10^4^ cells per well. A polarized cell monolayer was obtained by maintaining the culture in growth media for 12–14 days. Tissue culture media was changed every 48 h during polarization.

Prior to invasion experiments, 10 mL of an OD_600_ of 0.20 ± 0.02 bacterial suspension was prepared for both the *Salmonella* Typhimurium ATCC14028 and R2 in 0.9% saline. Suspensions were centrifuged at 4000× *g* (Eppendorf 5910R, Eppendorf, Hamburg, Germany) and the supernatant was removed. Pellets were resuspended in 1 mL 0.9% saline (corresponding to 10^9^ bacteria cells/mL). One hundred µL of each bacterial suspension was added separately to deionized water (maximum Salmonella invasion) and PTW types, vortexed, and incubated in PTW for 1 min.

Prior to the addition of bacteria, the polarized Caco-2 monolayer was washed three times with DMEM containing no supplements. Individual *Salmonella* strains treated with different PTW types were added separately to 48-well of the tissue culture trays containing polarized Caco-2 cells. Bacteria were added to a multiplicity of infection (MOI) of 100 into DMEM + 10% FBS. The presence of FBS inactivated the PTW. Bacteria were incubated with the cell monolayer for 1 h and were then removed by aspiration. Caco-2 cells were subsequently washed two times with DMEM containing no supplements. Five hundred μL DMEM containing 200 µg/mL gentamicin was then added to cells and incubated at 37 °C for 15 min. Media containing gentamicin was removed and the cell monolayers were washed three times with DMEM. Cells were lifted from the tissue culture tray by adding 0.25% trypsin and incubating at 37 °C for 10 min. Cells were then lysed in 250 μL of 1% Triton X-100 (Merck, Bayswater, Australia) at 37 °C for 30 min. Serial 10-fold dilutions were prepared and 100 μL of each dilution spread plated onto xylose lysine deoxycholate (XLD) agar, and incubated at 37 °C for 18 h. Bacterial colonies were enumerated, and the data are represented as Log_10_CFU/mL. Experiments were performed in triplicate over four independent experiments.

### 4.7. Culture, Biofilm Formation, and PTW Treatment of Salmonella Typhimurium

R2, R5, R7, and ATCC 14028 were streaked onto tryptone soya agar plates for 24 h at 37 °C. Single colonies were resuspended in 0.9% saline to a density of 1.0 ± 0.1 MFU, then diluted 1:15 in Luria Bertani broth (LB) without sodium chloride. For biofilm growth, 100 µL of each isolate was aliquoted into black-walled 96-well polystyrene plates (Corning Costar, Sigma Aldrich, Castle Hill, Australia) and incubated for 4 days statically at 22 °C. After 4 days incubation, broth was aspirated and the biofilms were washed twice with 130 µL 0.9% saline to remove planktonic bacteria, then left to dry for 5 min in the fume hood. LB, water control, and PTW were climatized to 22 °C before the addition to *Salmonella* Typhimurium. One hundred µL of LB (growth control; GC), water, PTW1 or PTW2 were added and incubated for 5 min at 37 °C. Experiments were performed in quintuplicate over four independent experiments.

### 4.8. Bacterial Viability Measured by Alamar Blue Assay

After PTW treatment, wells were aspirated and washed twice with 0.9% saline and dried again. Once dry, 100 µL of 10% *v*/*v* Alamar Blue solution in LB was added to each well, including to 5 empty wells as blanks, then incubated, protected from light on a rotating platform at 70 rpm (3D Gyratory Mixer, Ratek Instruments, Boronia, Australia) for 2 h at 22 °C. The fluorescence was measured using a FluoStar Optima plate reader (BMG Labtech, Ortenberg, Germany), with excitation band of 530 ± 5 nm and emission band of 590 ± 5 nm, gain of 1100, and with bottom optic. Bacterial killing relative to the GC was calculated using Equation (1), where F_T_, F_B_, and F_GC_ represent the fluorescence of treated cells, background fluorescence of 10% *v*/*v* Alamar Blue solution only, and the GC fluorescence, respectively. This was performed over four independent experiments. Since Equation (1) always normalized the GC to 100%, we independently analyzed the variance of the GC over four independent experiments to obtain the normalized standard deviation of the GC.

(1) $$Bacterial\;viability\;\left(\%\right)=100\left(\frac{F_{T}-F_{B}}{F_{GC}-F_{B}}\right)\;$$

### 4.9. Biofilm Formation Measured by Crystal Violet Assay

After PTW treatment, wells were aspirated and biofilms were immediately fixed with >99% methanol for 15 min. The methanol was then removed and the wells were allowed to air dry in the fume hood. Crystal violet (0.1% *w*/*v*) was then added to the wells and allowed to absorb into the biofilm for 15 min. The plates were then washed with deionized water three times (until no more crystal violet stain could be seen in the water). Plates were then air dried for several hours or overnight. Crystal violet-bound biofilm was solubilized with 30% acetic acid (Sigma Aldrich, Castle Hill, Australia) on a 3D gyratory mixer. After 15 min, solubilized crystal violet absorption was measured at 590 ± 5 nm using the FluoStar Optima plate reader. Biofilm formation relative to the GC was calculated similarly to Equation (1) using the absorbance values, but with 30% acetic acid as the blank. This was performed over four independent experiments. As with the Alamar Blue assay, we independently analyzed the variance of the GC over four independent experiments to obtain the normalized standard deviation of the GC absorbance.

### 4.10. Bioflux Experiment

The Bioflux system (Fluxion Biosciences, Oakland, CA, USA) was used to determine biofilm prevention under flow conditions as previously described [38]. All media were used at 25 °C. Results were obtained from 3 independent experiments. Bioflux plates were primed with 350 µL half-strength LB broth without NaCl and inoculated with 70 µL of an overnight culture of *Salmonella* Typhimurium ATCC 14028, adjusted to OD_600_ of 0.5. Following bacterial attachment for 30 min at 25 °C and no flow, bacteria were exposed to half-strength LB broth without NaCl for 18 h at 25 °C under steady nutrient flow (0.2 dyne/cm^2^), allowing for biofilm formation. Biofilms were treated with PTW for 20 min at 25 °C and 0.2 dyne/cm^2^ flow. After a washing step with 200 µL half-strength LB broth without NaCl for 10 min at 25 °C and 0.2 dyne/cm^2^ flow, Live/Dead staining (2 µL SYTO 9 and 1 µL propidium iodide in 10 mL half-strength LB broth without NaCl) was added first for 5 min at 25 °C and 0.2 dyne/cm^2^ flow, then for 30 min without flow. Biofilm growth was monitored through brightfield microscopy (magnification 20×) and bacterial viability were visualized through green/red microscopy channels. Images were automatically taken every 15 min throughout the entire experiment. Following another washing step with half-strength LB broth without NaCl, biofilms were incubated for 3 days at 25 °C without flow to observe potential bacterial regrowth.

### 4.11. Statistics

Data were analyzed using GraphPad Prism version 9.5.1 (GraphPad Software, Boston, MA, USA). Data were tested for normality using the Shapiro—Wilk normality test and are represented by the mean ± standard deviation. Effect of PTW on bacterial invasion, biofilm, and cell viability was tested using a repeated measures one-way analysis of variance with Šídák’s multiple comparisons test for parametric data or Kruskal—Wallis with Dunn’s multiple comparison test for non-parametric data. An alpha value of 0.05 was considered statistically significant.

## 5. Conclusions

The results presented here demonstrate the efficacy of specific PTW types on controlling *Salmonella* and highlight their potential as a method to control the bacteria in the food supply chain. Given the industrial chemical-free nature, PTW has several advantages over currently used food sanitizers. Reactions of PTW mostly result in producing water and low levels of highly soluble nitrate [16]. As such, PTW does not leave harmful chemical waste residue during its use. As accessibility to plasma treatment becomes more widespread, PTW production on-site and to-demand scale will be an economical and environmentally friendly way to produce potent, broad-spectrum antimicrobial solutions, as opposed to relying on industrial (usually fossil fuel-based) disinfectants, detergents, and oxidizing substances. Implementation into commercial use needs to consider contact time, method, and what long-term effect PTW solutions may have on equipment and the sensory quality of food. Future research looking into the mechanism of action, ultrastructural effects of PTW on food quality, shelf-life and safety, as well as other food science relevant experiments is warranted.

## Figures and Tables

**Table 1:** Physiochemical properties of plasma-treated water types.

ID	Resistivity (μΩ × m)	Conductivity (S/m)	ORP (mV)
PTW1	2200	4.55 × 10^−4^	626
PTW2	806	1.24 × 10^−4^	582
PTW3	1122	8.91 × 10^−4^	594
PTW4	3890	2.57 × 10^−4^	593
PTW5	826	1.21 × 10^−4^	592

ID: identity, ORP: oxidoreductive potential.

## Data Availability

All data generated or analyzed in this study are presented in this manuscript.

## Supplementary Materials

The following supporting information can be downloaded at: https://www.mdpi.com/article/10.3390/antibiotics12091371/s1, Video S1: Shrinking and partial detachment of 18 h-old *Salmonella* Typhimurium ATCC 14028 biofilms after 5 min of treatment with PTW1, Video S2: Shrinking and partial detachment of 18 h-old *Salmonella* Typhimurium ATCC 14028 biofilms after 5 min of treatment with PTW2.
